# Biventricular Strain Imaging with Cardiac MRI in Genotyped and Histology Validated Amyloid Cardiomyopathy

**DOI:** 10.3390/cardiogenetics11030011

**Published:** 2021-06-30

**Authors:** Abhinay Reddy, Vasvi Singh, Badri Karthikeyan, Leyi Jiang, Silva Kristo, Sharma Kattel, Ram Amuthan, Saraswati Pokharel, Umesh C. Sharma

**Affiliations:** 1 Department of Medicine, Division of Cardiology, Jacob’s School of Medicine and Biomedical Sciences, Buffalo, NY 14203, USA; 2 Department of Radiology, Division of Nuclear Medicine, Brigham and Women’s Hospital and Harvard Medical School, Boston, MA 02115, USA; 3 Department of Pathology and Laboratory Medicine, Roswell Park Comprehensive Cancer Center, Buffalo, NY 14203, USA; 4 Department of Medicine, Division of Cardiology, Yale School of Medicine, New Haven, CT 06510, USA

**Keywords:** amyloidosis, cardiac MRI, endomyocardial biopsy, strain imaging

## Abstract

Cardiac amyloidosis (CA) is a common and potentially fatal infiltrative cardiomyopathy. Contrast-enhanced cardiac MRI (CMR) is used as a diagnostic tool. However, utility of CMR for the comprehensive analysis of biventricular strains and strain rates is not reported as extensively as echocardiography. In addition, RV strain analysis using CMR has not been described previously.

**Objectives:**

We sought to study the global and regional indices of biventricular strain and strain rates in endomyocardial biopsy (EMB)-proven, genotyped cases of CA.

**Methods:**

A database of 80 EMBs was curated from 2012 to 2019 based on histology. A total of 19 EMBs positive for CA were subjected to further tissue-characterization with histology, and compared with four normal biopsy specimens. Samples were genotyped for ATTR- or AL-subtypes. Five patients, with both echocardiography and contrast-enhanced CMR performed 72-h apart, were subjected to comprehensive analysis of biventricular strain and strain-rates.

**Results:**

Histology confirmed that the selected samples were indeed positive for cardiac amyloid. Echocardiography showed reduced global and regional left-ventricular (LV) longitudinal strain indices. CMR with tissue-characterization of LV showed global reductions in circumferential, radial and longitudinal strains and strain-rates, following a general trend with the echocardiographic findings. The basal right-ventricular (RV) segments had reduced circumferential strains with no changes in longitudinal strain.

**Conclusions:**

In addition to providing a clinical diagnosis of CA based on contrast clearance-dynamics, CMR can be a potent tool for accurate functional assessment of global and regional changes in strain and strain-rates involving both LV and RV. Further studies are warranted to validate and curate the strain imaging capacity of CMR in CA.

## 1. Introduction

Cardiac amyloidosis (CA) has poor prognosis unless identified and treated early. On histology, CA shows infiltration and deposition of abnormal non-contractile extracellular proteins within myocardial tissue [1]. This process leads to initial diastolic dysfunction and ultimately systolic dysfunction, often involving both left and right ventricles [2]. Recently, there have been significant advances in the multimodality imaging of CA, and the disease is being increasingly recognized as a cause of heart failure [2]. CA presents in two distinct forms: ATTR amyloidosis, which is due to deposition of transthyretin in myocardium, and AL amyloidosis, which is a plasma cell dyscrasia distinct from multiple myeloma [2,3].

Recent clinical trials have demonstrated that CA is a treatable disease [4]. The specificity of diagnosing ATTR-CA with PYP scan is very high, whereas AL-CA can be ascertained by serum and urine tests. However, current imaging modalities for myocardial functional evaluation of CA are limited: echocardiography is the primary means of noninvasively diagnosing cardiac amyloidosis, with findings including speckling and strain imaging lending confidence to the diagnosis [5]. However, echocardiography is known to be operator-, equipment- and reader-dependent, leading to variability [6]. In addition, acoustic windows are easily affected by patient anatomy. As such, the gold standard for diagnosis of cardiac amyloidosis is an invasive endocardial biopsy, both to diagnose and correctly genotype the underlying pathology [7].

Cardiac MRI (CMR) has been shown to play an increasingly important role in the detection of cardiac amyloidosis. Contrast-enhanced CMR can determine the myocardial amyloid burden [8], particularly as the infiltrative process can advance prior to reduction in LVEF [9]. The sensitivity and specificity for characteristic late gadolinium enhancement (LGE) to diagnose amyloidosis by CMR has been shown to be 85% and 92%, respectively [10]. Echocardiography is not able to perform tissue characterization in the same manner as CMR, but does reliably show abnormal strain in CA [11]. CMR also has been shown to have increasing utility in prognostication of cardiac amyloidosis, particularly CMR-derived indexed stroke volume and wall excursions [9,12].

In this study, we aimed to demonstrate the feasibility of biventricular strain imaging using CMR in EMB proven cardiac amyloid, illustrate the parallels of CMR strain imaging with echocardiographic strain imaging, and provide an initial foray into CMR based evaluation of right ventricular strain [13].

## 2. Methods

### 2.1. Study Design

This is a multimodality retrospective feasibility study in a select group of patients with biopsy-proven cardiac amyloidosis. All patients were selected from a single, tertiary care center, Buffalo General Hospital (BGH) and Gates Vascular Institute (GVI) in Buffalo, New York. The study was approved by the University at Buffalo Institutional Review Board. An institutional database of 80 endomyocardial biopsies (EMB) from 2012 through 2019 was reviewed to identify 19 patients with cardiac amyloidosis on histology, with corresponding controls. From this cohort, 5 patients and corresponding controls were further identified to have echocardiography and contrast enhanced CMR done 72 h apart.

### 2.2. Histopathological Analysis and Genotyping of Endomyocardial Biopsies

EMBs were initially evaluated at BGH by hematoxylin–eosin (H&E) staining. Tissue sections showing homogeneous eosinophilic material on H&E were subjected to Congo red staining. Briefly, 10-micron paraffin embedded sections were deparaffinized, hydrated, and stained with Congo red. These sections were counterstained with hematoxylin, washed, dehydrated in 100% alcohol, cleared in xylene and mounted. Amyloid deposition was determined with bright field microscopy under polarized light. Amyloid typing was performed with liquid chromatography tandem mass spectrometry (LC MS/MS) on peptide extracted from Congo-red positive microdissected areas of the paraffin embedded specimens at Mayo Clinic (Rochester, MN, USA), and confirmatory genetic testing was performed for transthyretin cardiac amyloidosis (ATTR-CA).

### 2.3. Contrast-Enhanced Cardiac MRI Protocol

A GE 1.5-T scanner with technical parameters recommended by the manufacturer was used. After acquiring the localizer/scout images in coronal, sagittal and axial planes, fast spin-echo (FSE) axial slices, short-axis and two, three, and four-chamber steady-state free precession (SSFP) sequences, T2-weighted triple-inversion recovery images and T1-weighted FSE sequence were obtained. Then, delayed enhanced images were obtained 2–10 min after intravenous (IV) gadolinium injection (Omniscan 0.1 mmol/kg) using previously validated inversion recovery pulse sequence. The TI Cardiac function and strain imaging was performed by using Segment version 3.1 R8123 (http://segment.heiberg.se accessed on 15 June 2020), as described previously by our group [14-18].

### 2.4. Strain Analysis

#### Echocardiography-Based Approach:

Studies were uploaded to TomTec software module (TOMTEC USA, Chicago, IL, USA). Two-dimensional speckle tracking echocardiography was done using TomTec AutoSTRAIN, Version; Image-Com5 5.5.4.467461 to determine segmental longitudinal strain of all the visualized left ventricle segments. The endocardium was visualized and traced during end-systole and end-diastole. The software automatically tracked the region-of-interest during the cardiac cycle. The magnitude of deformation was used to generate the strain curves.

#### Cardiac MRI-Based Approach:

Strain analysis was conducted on amyloidosis patients using principles from finite strain theory, as described previously [14]. Cardiomyocyte contraction was evaluated by using one-dimensional Lagrangian strains (*ε*) along the circumferential, radial and longitudinal directions, which are defined by the following formula [19]:

(1) $$\varepsilon =\frac{L-L_{0}}{L_{0}}$$

where *L* is the length of the myocardial segment and *L*_0_ is the original length at end diastole. Cardiac contraction and relaxation can be measured using Eulerian strain rates (SR), which are based on using the myocardial velocity gradient as shown below [19]:

(2) $$SR=\frac{v_{2}-v_{1}}{L}=\frac{1}{L}\frac{dL}{dt}=\frac{1}{\varepsilon +1}\frac{d\varepsilon }{dt}$$

where *v*_1_ and *v*_2_ are myocardial velocities. Circumferential and radial strains were measured by using three short-axis slices at the level of basal, midventricular and apical regions. Longitudinal strains were evaluated by using 2, 3 and 4-chamber long-axis images. Strains were determined by drawing the left ventricular (LV) and right ventricular (RV) borders at end diastole, after which the analysis module created the contours automatically. Quality control was achieved by manual adjustment of the initial contour of end-diastolic image. Quantitative analyses for strains were performed at end-systole, while quantitative analyses for the strain rate were done at peak-systole (SR_S_) and peak-diastole (SR_D_), which represent the two peaks seen in the strain curves, respectively [14]. Both LV and RV strains and strain rates were evaluated globally, as well as in basal, midventricular, apical and septal regions. Additionally, RV lateral strains and strain rates were also measured to evaluate RV free wall deformation.

### 2.5. Statistical Methods

Quantitative findings were summarized within groups, using the mean and standard error of the mean (SEM). Quantitative regional comparisons were done using unpaired Student’s *t*-tests and Analysis of Variance (ANOVA). All tests were 2-sided, and *p* < 0.05 was considered significant.

## 3. Results

The mean age of 19 subjects with endomyocardial biopsy proven cardiac amyloidosis was 71 years, with a sex distribution 74% males. Out of 19 subjects, 10 had ATTR and 8 had AL cardiac amyloidosis, with one patient having an insufficient sample for subtyping (Supplementary Table S1).

### 3.1. Histopathology of EMB Samples

**Figure 1.**
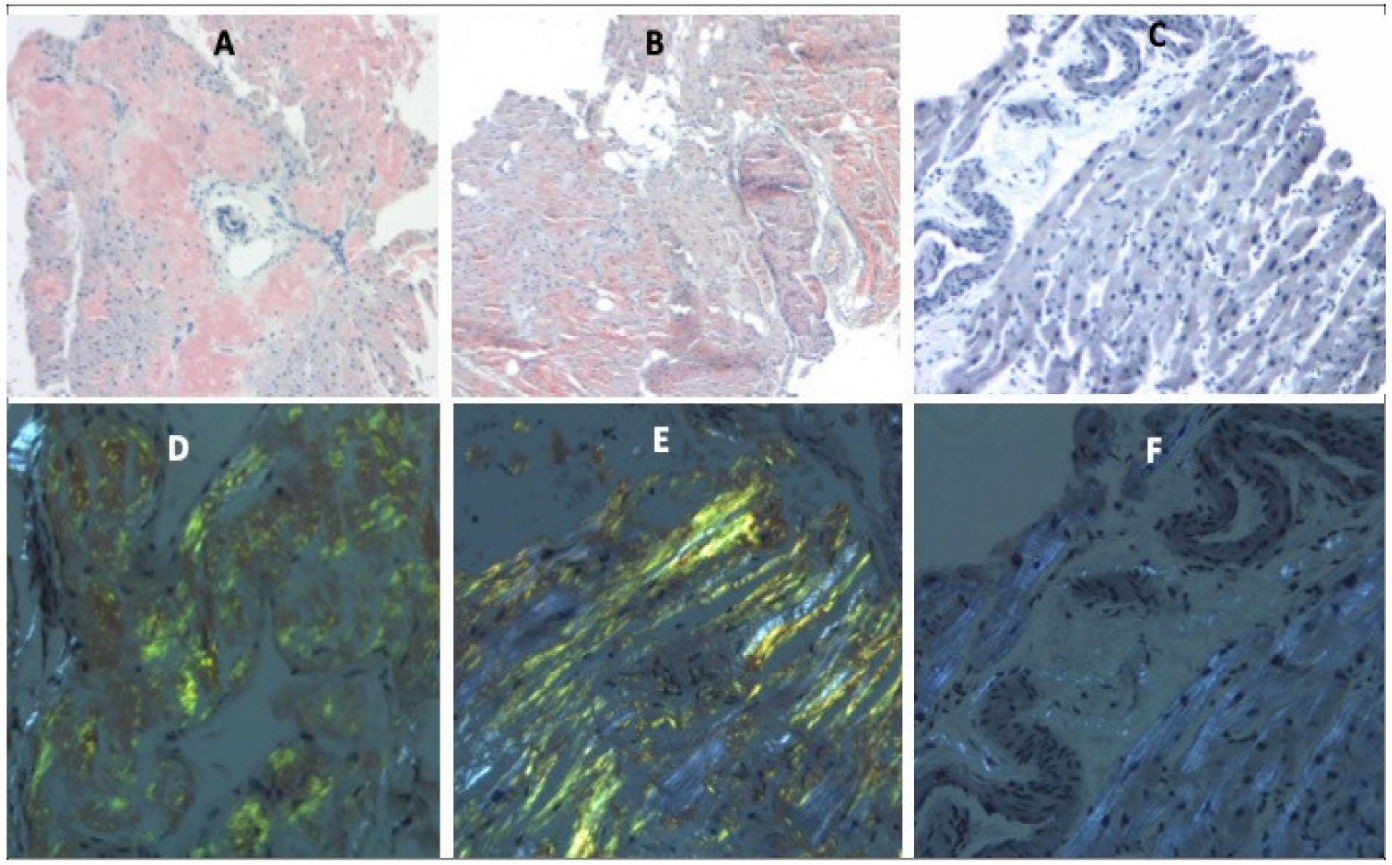
Bright field microscopy (**A–C**) and polarized light (**D–F**) display of Congo red-stained myocardial sections. Amyloid deposits are orange-red on Congo red staining with bright field microscopy and display apple-green birefringence under polarized light. (**A**,**D**) ATTR amyloid, (**B**,**E**) AL amyloid, and (**C**,**F**) Control myocardium.

LC MS/MS analyses sub-classified the amyloid to ATTR and AL subtypes. Congo red-stained deposits are orange-red with bright field microscopy and display apple-green birefringence under polarized light. (Figure 1).

### 3.2. Echocardiographic Assessment of Left Ventricular (LV) Longitudinal Strains

**Figure 2.**
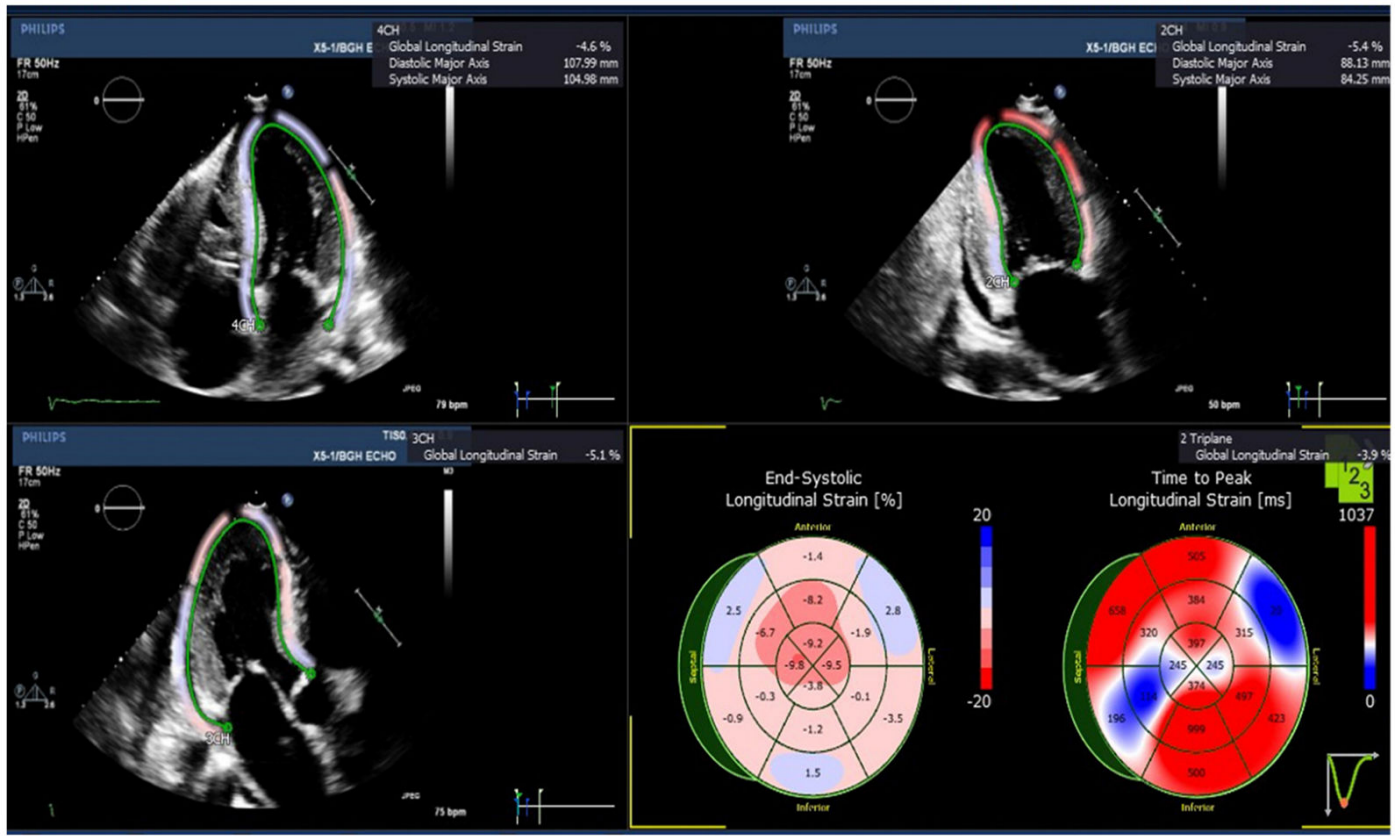
Left ventricular (LV) longitudinal strain imaging of cardiac amyloidosis using speckle tracking echocardiography. The basal segments are noted to exhibit dyskinetic motion, as observed by the outward movement of the basal inferior, basal anteroseptal and basal anterolateral segments. The apical segments are more contractile, but still relatively hypokinetic.

A summary of comparative differences in the longitudinal strains measured by speckle tracking echocardiography is provided in Table 1. Representative echocardiograms of cardiac amyloidosis are shown in Figure 2. The global longitudinal strain (GLS) of the LV with cardiac amyloidosis was −7.96 ± 1.45% when compared to the GLS of a LV with normal group, which was −19.44 ± 0.33% (*p* < 0.01). There was significant reduction in LV segmental longitudinal strain of all the LV segments except in the basal inferoseptal, apical-inferior, apical-anterior and apical-lateral segments. The longitudinal strain of the amyloid apical septal myocardium was −14.50 ± 2.83%, which was lower than that of the normal apical septal myocardium at −20.86 ± 1.29%.

### 3.3. Cardiac MRI Assessment of Left Ventricular (LV) Deformation

**Figure 3.**
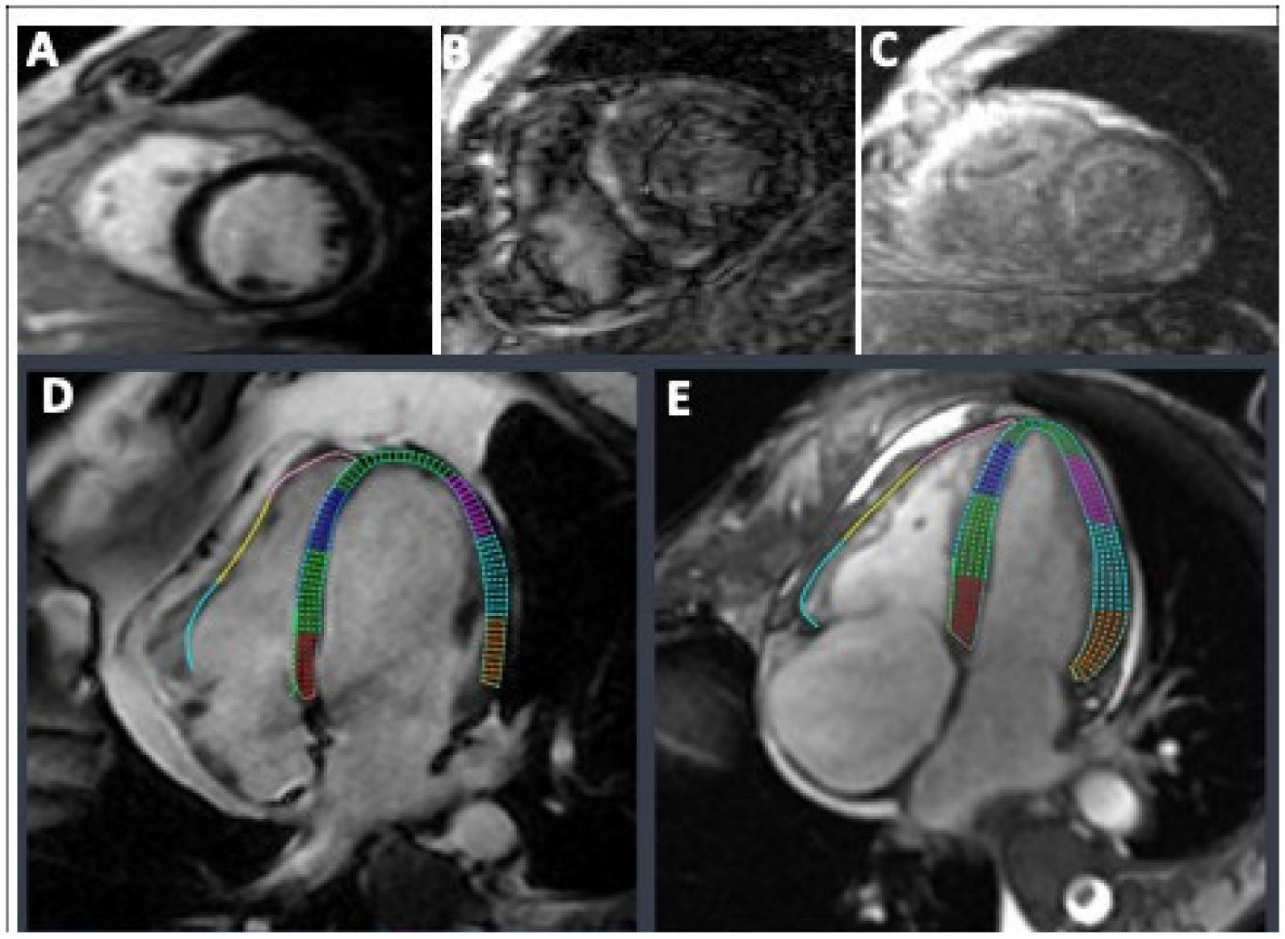
Cardiac MRI (CMR) visualization of cardiac amyloidosis using late gadolinium enhancement (LGE) and strain imaging. (**A–C**) Lack of myocardial nulling and characteristic speckled appearance are noted after gadolinium injection in cardiac amyloidosis. (**A**) Representative LGE-CMR short-axis image of a normal patient. (**B**) Representative LGE-CMR short-axis image of an ATTR amyloidosis patient. (**C**) Representative LGE-CMR short-axis image of an AL amyloidosis patient. (**D**,**E**) Visualization of left and right ventricular longitudinal strains under 4 chamber (4CH) long-axis view. (**D**) Representative 4CH-CMR image of a normal patient. (**E**) Representative 4CH-CMR image of an amyloidosis patient.

**Figure 4.**
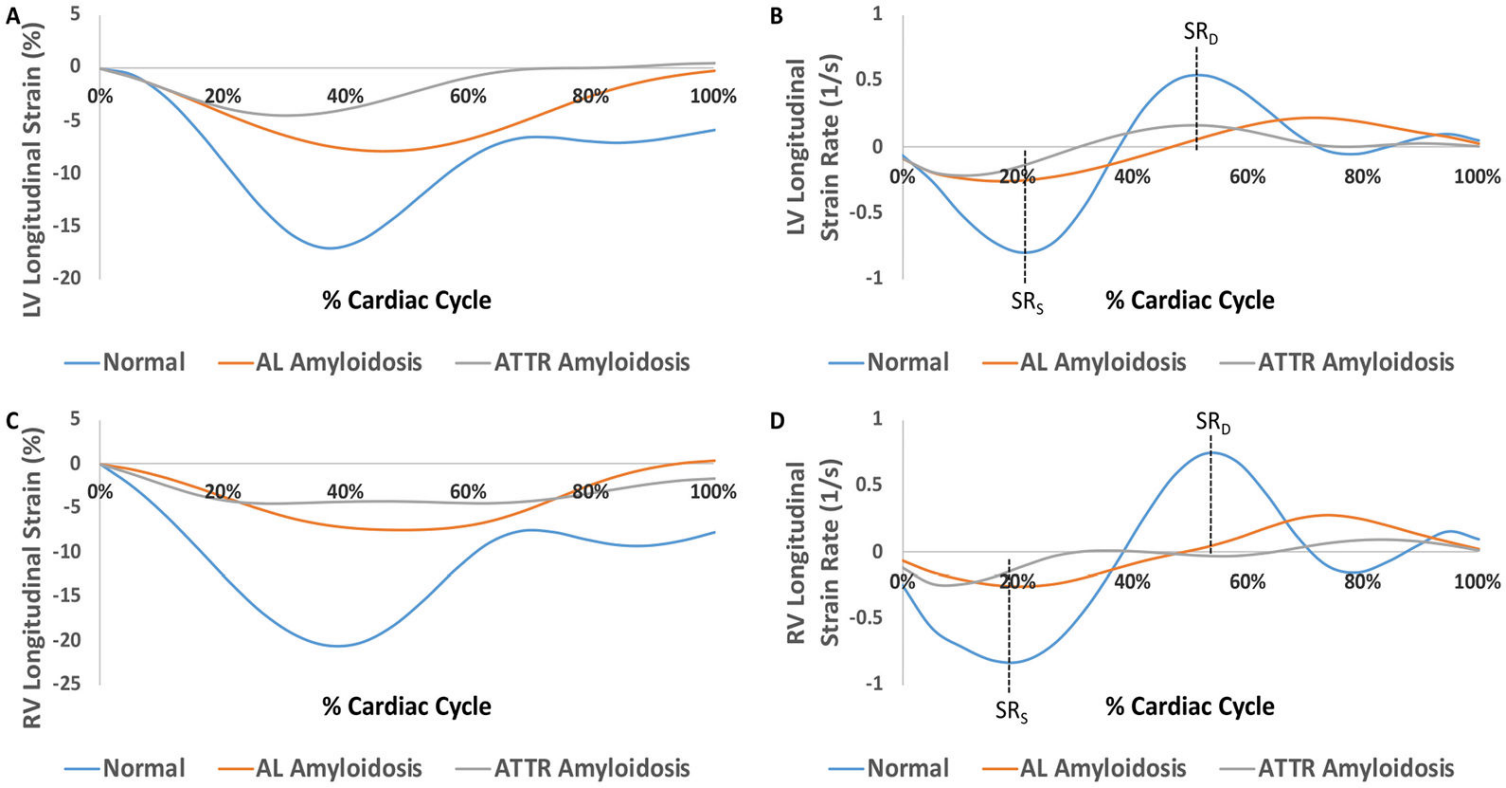
Strain curves illustrating left ventricular (LV) and right ventricular (RV) changes in global longitudinal strain and strain rate over the course of one cardiac cycle. Representative strain curves were used from a normal patient, an AL amyloidosis patient, and an ATTR amyloidosis patient. (**A**) LV longitudinal strain curves; (**B**) LV longitudinal strain rate curves; (**C**) RV longitudinal strain curves; (**D**) RV longitudinal strain rate curves.

First, the amyloidosis pattern was visualized based on diffuse myocardial late enhancement affecting the entire ventricle with a pathological gadolinium clearance (Figure 3). Strain analysis was conducted on five biopsy-proven cardiac amyloidosis cases with five corresponding age-matched controls. The control group exhibited preserved strain patterns across the circumferential, radial and longitudinal axes in all myocardial segments (basal, mid, apical and global) when compared with controls and previously published values [20]. Representative curves illustrating LV changes in longitudinal strain and strain rates for a normal patient, an AL amyloidosis patient, and an ATTR amyloidosis patient are shown in Figure 4A,B.

#### 3.3.1. LV Circumferential, Radial and Longitudinal Strains

**Figure 5.**
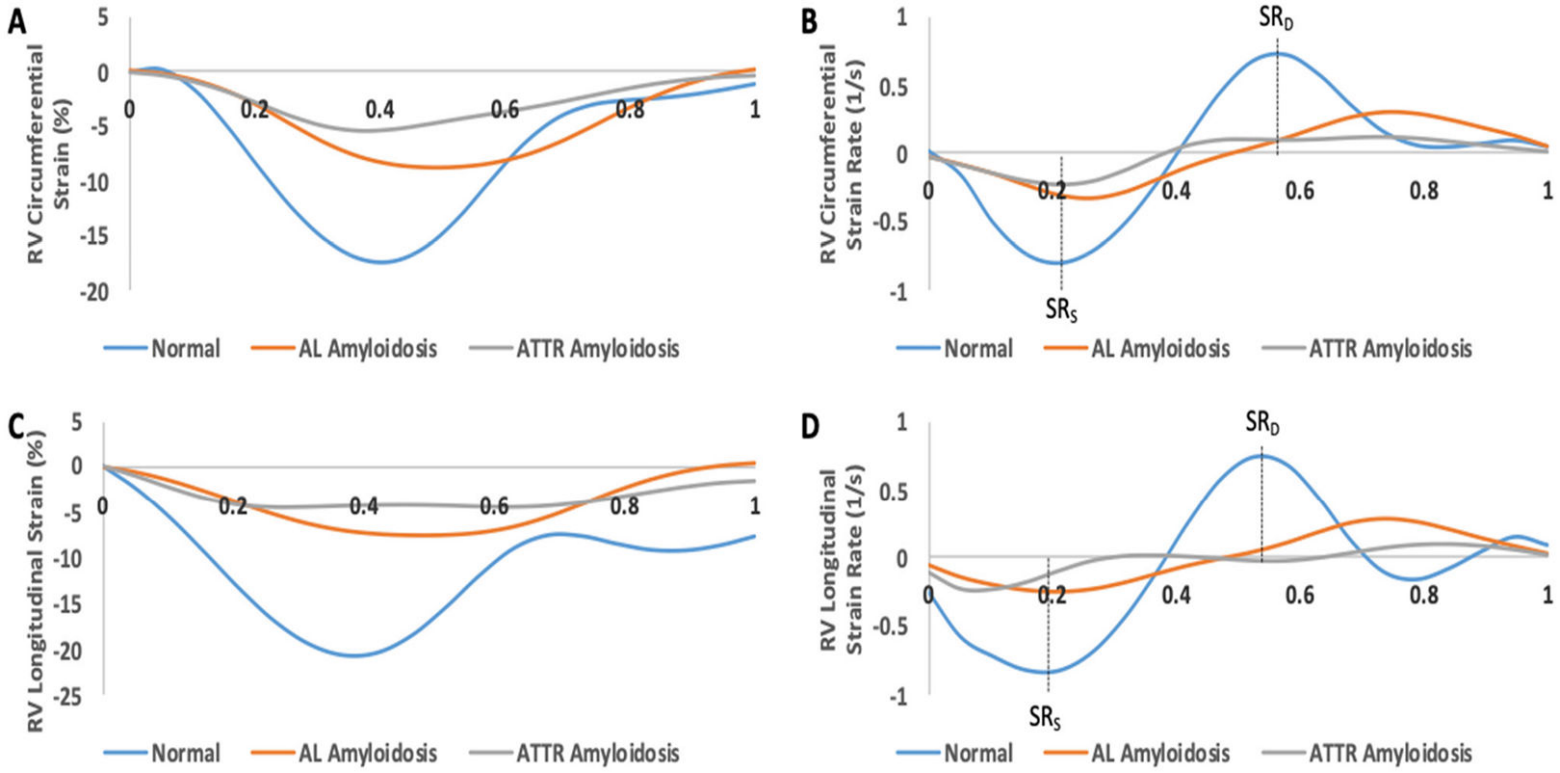
Strain curves illustrating right ventricular (RV) global changes in strain and strain rate over the course of one cardiac cycle. Representative strain curves were used from a normal patient, an AL amyloidosis patient, and an ATTR amyloidosis patient. (**A**) RV circumferential strain curves; (**B**) RV circumferential strain rate curves; (**C**) RV longitudinal strain curves; (**D**) RV longitudinal strain rate curves. SR_S_ = peak systolic strain rate; SR_D_ = peak diastolic strain rate.

Compared to controls, amyloidosis patients had exhibited significantly reduced circumferential, radial and longitudinal strains in the basal, midventricular, and apical segments (Figure 5, Table 2). These strain parameters in the LV septum were also significantly lower in amyloidosis patients. Amyloidosis patients also had an overall reduction in global circumferential strain (Table 2).

#### 3.3.2. LV Strain Rates

Compared to normal patients, amyloidosis patients had reduced peak-systolic and diastolic circumferential strain rates in the basal and midventricular segments. These patients had an overall reduction in global peak systolic and diastolic circumferential strain rates (Table 2). The comparison of radial and longitudinal strain rates in different myocardial territories measured during both end-systole and end-diastole are shown in Table 2.

### 3.4. Cardiac MRI Assessment of Right Ventricular (RV) Deformation

RV strains were calculated in a fashion similar to that of LV strains, but radial values could not be reported due to the physical structure of the RV. The basal right-ventricular (RV) segments had reduced circumferential strains with no changes in longitudinal strain. The RV septal segments showed significant reductions in circumferential and longitudinal strains. Representative curves and quantitative comparison illustrating RV changes in circumferential and longitudinal strain and strain rates for a normal patient, an AL amyloidosis patient, and an ATTR amyloidosis patient are shown in Figures 4C,D and 5 and Table 3.

## 4. Discussion

Our study further underscores the ability of CMR to identify patients with CA. While it is known that CMR is clinically indicated as a diagnostic modality to identify CA using LGE, we describe another diagnostic dimension of CMR that exhibits parallel trends with echocardiography: strain imaging. Our findings suggest that detection of abnormal gadolinium clearance kinetics and quantification of global and regional deformation by contrast-enhanced CMR can be complementary diagnostic tools for cardiac amyloid.

When evaluating CA, echocardiography has long been the standard for non-invasive imaging techniques, with recent advances including strain rate imaging and speckle tracking improving its efficacy [21,22]. Echocardiography with high frame rates is known to be effective in strain imaging. However, echocardiography may have challenges with image quality, which can be a reflection of a patient’s body habitus, technical limitations or user variability (for instance, appropriate placement of the probe to minimize the angle between the beam and the LV wall if tissue doppler-based strain imaging techniques are employed) [23,24]. In addition, the LV apex is typically omitted on echocardiograms due to image shortening [25].

Another diagnostic pathway utilizes CMR. Technological improvements have greatly improved its efficacy in measuring LVEF and ventricular volumes [26]. CMR is mostly not affected by body habitus, and standardized protocols have been developed for comprehensive myocardial imaging. CMR has the benefit of tissue characterization, which cannot be completed using ultrasound-based techniques [27]. In this study, we were able to analyze EMB proven cardiac amyloidosis with CMR, and focused on the often-underutilized strain imaging capability of CMR, as tissue characterization with LGE is well described in the literature. EMB proven CA was essential to directly link CMR based strain imaging to CA, in order to remove the risk of false-positive and false-negative diagnoses, which can occur when solely relying on non-invasive imaging.

The results from our CMR analysis have shown mild-apical sparing in LV peak systolic circumferential strain rate. Instead, there was a consistent reduction in circumferential, radial and longitudinal strains in the basal, midventricular and apical regions. These results echoed the trends seen in echocardiographic based strain imaging; statistical analysis, namely regression analysis, could not be conducted during this study due to its small sample size; this should be explored in future larger, prospective studies. An interesting finding is that both the LV and RV septal segments showed significant reductions in systolic strain and strain rates, which suggests that cardiac amyloidosis might affect the contractility of the interventricular septum. A repository of standardized CMR strain values must be curated, and validated by established strain imaging echocardiography, to provide clinicians with a reference by which to characterize myocardial strain using CMR in patients with amyloid myocardium [28,29]. For the purposes of this study, our CA samples and corresponding analyses were compared to controls without any major structural or infiltrative heart diseases; in addition, there is no consensus for normal strain rate values even when using speckle tracking echocardiography [8].

When assessing the RV, circumferential and longitudinal strains were analyzed, as the thin RV myocardium does not make assessing radial strain possible. Apical sparing was observed in the RV systolic circumferential strain, while RV systolic longitudinal strain was reduced in the apical region. Both the RV circumferential and longitudinal strains did not show significant differences in the midventricular and lateral RV segments, which could suggest possible sparing of these regions. It is known that RV involvement is common in CA. Current literature reports, however, have only recently studied RV-LGE, but these have not yet been validated as a prognostic tool [30,31]. In order to provide a more complete diagnostic picture for CA, strain imaging and LGE both need to be taken into consideration when using non-invasive imaging methods such as CMR [31]. To date, there has only been minimal study of the RV using strain imaging techniques, both with echocardiography and CMR.

Strain rate analysis also shows promise in the diagnosis of CA. Early diastolic LV strain rate was determined to be an independent predictor of outcomes in patients with preserved EF [32]. However, there is no current literature that describes changes in RV strain rate. Our study shows a mild apical sparing in RV peak-diastolic circumferential strain rate, but this apical sparing pattern is not seen in other RV strain rates. Previous studies have primarily looked at LV strain while neglecting to determine systolic strain rate [28]. However, further larger studies need to be done to validate these results.

### Limitations:

CMR has some limitations and may not be the most suitable imaging modality particularly in patients with renal dysfunction and presence of MRI-non-conditional devices. However, the advent of new contrast agents that are substantially less nephrotoxic and the shift in implantable devices to those that are MRI compatible will allow for more broader application of CMR to a larger population of patients [33]. Presently, acquisition time of CMR images is far longer than conventional echocardiography, although with advances in MRI technology these shortcomings should improve. Our study has a few other limitations which can be overcome with future research. This is a retrospective study with a small sample size which aims to illustrate the feasibility of using CMR to determine myocardial strain in CA; as a result, determining sensitivity and specificity of myocardial strain is difficult. Regression analysis between echocardiography and CMR is also limited due to the small sample size; as a result, complete validation of our CMR based strain with echocardiography has not been done and rather, general trends have been reported. In addition, delineation between ATTR and AL amyloidosis using CMR strain imaging was not conducted due to the small sample size.

## 5. Conclusions

CMR can be a powerful non-invasive diagnostic modality for myocardial tissue characterization and strain imaging, providing a potential one-stop shop for non-invasive imaging in infiltrative cardiac disease. However, further large prospective studies are needed to validate strain findings with parallel modalities such as echocardiography and apply them to a more inclusive patient population.

## Supplementary Material

Supplementary Material

## Figures and Tables

**Table 1.** Left ventricle longitudinal strain characteristics of amyloid patients compared to normal subjects using speckle tracking echocardiography.

GLS (%), Mean (SEM)	Amyloid Patients (*n* = 5)	Normal Subjects (*n* = 8)
GLS, triplane	−7.96 (1.45) *	−19.44 (0.33)
GLS, apical four chamber	−9.10 (1.42) *	−21.51 (0.62)
GLS, apical two chamber	−8.12 (1.72) *	−19.35 (0.77)
GLS, apical three chamber	−8.0 (1.11) *	−18.45 (0.64)
Basal anterior	−4.54 (1.60) *	−26.65 (1.57)
Basal anteroseptal	−2.42 (2.10) *	−15.78 (3.38)
Basal inferoseptal	−5.96 (1.66)	−16.85 (4.61)
Basal inferior	−3.44 (2.17) *	−20.26 (3.65)
Basal inferolateral	−8.5 (2.92) *	−32.54 (3.13)
Basal anterolateral	−5.88 (3.09) *	−34.56 (2.77)
Mid anterior	−6.62 (1.10) *	−15.23 (1.80)
Mid anteroseptal	−6.88 (1.94) *	−16.83 (2.50)
Mid inferoseptal	−5.74 (1.97) *	−18.15 (2.34)
Mid inferior	−6.40 (1.5) *	−16.15 (2.20)
Mid inferolateral	−1.96 (1.91) *	−10.74 (1.97)
Mid anterolateral	−3.30 (1.33) *	−16.23 (2.11)
Apical anterior	−12.94 (4.27)	−15.95 (2.49)
Apical septal	−14.50 (2.83) *	−20.86 (1.29)
Apical inferior	−11.76 (3.60)	−19.20 (1.70)
Apical lateral	−13.32 (1.55)	−17.23 (2.66)

Abbreviations: GLS = Global longitudinal strain; SEM = Standard error of mean

(*) indicates *p* < 0.05.

**Table 2.** Left ventricular (LV) strain and strain rate characteristics of amyloidosis patients compared to controls using cardiac MRI.

Parameters	Controls (N = 5)	Amyloidosis (N = 5)	*p* Value
***LV Systolic Circumferential Strain (%)***			
Basal	−18.133 ± 1.742	−6.063 ± 1.739	0.001 *
Midventricular	−17.014 ± 0.987	−8.928 ± 1.545	0.002 *
Apical	−20.159 ± 2.630	−9.944 ± 2.645	0.025 *
Septal	−19.778 ± 1.756	−9.248 ± 1.936	0.004 *
Global	−18.220 ± 1.535	−8.108 ± 1.734	0.002 *
***LV Systolic Radial Strain (%)***			
Basal	41.404 ± 5.938	10.347 ± 4.655	0.003 *
Midventricular	40.361 ± 1.523	17.909 ± 4.964	0.003 *
Apical	36.224 ± 4.916	12.045 ± 7.220	0.024 *
Septal	30.588 ± 2.199	5.628 ± 2.657	<0.001 *
Global	39.718 ± 3.533	13.607 ± 5.138	0.003 *
***LV Systolic Longitudinal Strain (%)***			
Basal	−18.525 ± 0.735	−8.859 ± 2.741	0.009 *
Midventricular	−14.884 ± 1.120	−6.496 ± 1.532	0.002 *
Apical	−10.998 ± 1.367	−3.543 ± 0.965	0.002 *
Septal	−14.036 ± 1.276	−7.395 ± 1.437	0.009 *
Global	−15.278 ± 0.929	−6.644 ± 1.398	0.001 *
***LV Peak Systolic Circumferential Strain Rate (s^−1^)***			
Basal	−0.793 ± 0.091	−0.246 ± 0.080	0.002 *
Midventricular	−0.756 ± 0.077	−0.348 ± 0.069	0.004 *
Apical	−1.027 ± 0.256	−0.432 ± 0.128	0.071
Septal	−0.868 ± 0.102	−0.374 ± 0.086	0.006 *
Global	−0.837 ± 0.122	−0.331 ± 0.080	0.009 *
***LV Peak Systolic Radial Strain Rate (s^−1^)***			
Basal	1.278 ± 0.210	0.452 ± 0.175	0.017 *
Midventricular	1.276 ± 0.028	0.619 ± 0.152	0.003 *
Apical	1.077 ± 0.133	0.403 ± 0.223	0.032 *
Septal	0.971 ± 0.105	0.243 ± 0.069	<0.001 *
Global	1.227 ± 0.111	0.502 ± 0.172	0.008 *
***LV Peak Systolic Longitudinal Strain Rate (s^−1^)***			
Basal	−0.740 ± 0.068	−0.390 ± 0.150	0.066
Midventricular	−0.645 ± 0.060	−0.258 ± 0.051	0.001 *
Apical	−0.498 ± 0.088	−0.141 ± 0.048	0.007 *
Septal	−0.561 ± 0.074	−0.305 ± 0.061	0.029 *
Global	−0.644 ± 0.062	−0.278 ± 0.065	0.004 *
***LV Peak Diastolic Circumferential Strain Rate (s^−1^)***			
Basal	0.689 ± 0.072	0.180 ± 0.042	<0.001 *
Midventricular	0.678 ± 0.044	0.271 ± 0.061	0.001 *
Apical	0.803 ± 0.172	0.346 ± 0.095	0.049 *
Septal	0.812 ± 0.104	0.286 ± 0.063	0.002 *
Global	0.713 ± 0.072	0.255 ± 0.046	0.001 *
***LV Peak Diastolic Radial Strain Rate (s^−1^)***			
Basal	−1.294 ± 0.149	−0.317 ± 0.082	<0.001 *
Midventricular	−1.350 ± 0.215	−0.494 ± 0.118	0.008 *
Apical	−1.264 ± 0.167	−0.415 ± 0.210	0.013 *
Septal	−1.112 ± 0.148	−0.290 ± 0.035	0.001 *
Global	−1.307 ± 0.137	−0.408 ± 0.109	0.001 *
***LV Peak Diastolic Longitudinal Strain Rate (s^−1^)***			
Basal	0.598 ± 0.059	0.296 ± 0.076	0.014 *
Midventricular	0.504 ± 0.072	0.166 ± 0.030	0.002 *
Apical	0.380 ± 0.068	0.114 ± 0.044	0.011 *
Septal	0.461 ± 0.081	0.211 ± 0.025	0.019 *
Global	0.508 ± 0.059	0.202 ± 0.026	0.001 *

Values are presented as mean ± standard error of mean (SEM).

(*) indicates *p* value < 0.05 for amyloidosis patients compared to controls.

**Table 3.** Right ventricular (RV) strain and strain rate characteristics of amyloidosis patients compared to controls using cardiac MRI.

Parameters	Controls (N = 5)	Amyloidosis (N = 5)	*p* Value
***RV Systolic Circumferential Strain (%)***			
Basal	−11.376 ± 1.208	−3.564 ± 0.949	0.001 *
Midventricular	−10.269 ± 0.509	−9.079 ± 1.482	0.469
Apical	−14.775 ± 3.606	−8.064 ± 1.815	0.135
Lateral	−8.463 ± 2.193	−6.999 ± 1.210	0.575
Septal	−15.817 ± 1.715	−6.805 ± 1.433	0.004 *
Global	−12.140 ± 1.606	−6.902 ± 1.258	0.033 *
***RV Systolic Longitudinal Strain (%)***			
Basal	−17.883 ± 1.621	−12.600 ± 3.009	0.161
Midventricular	−16.811 ± 2.586	−10.758 ± 2.433	0.127
Apical	−12.337 ± 3.113	−2.168 ± 1.414	0.018 *
Lateral	−19.283 ± 3.066	−11.309 ± 2.138	0.065
Septal	−12.070 ± 1.395	−5.709 ± 1.119	0.007 *
Global	−15.677 ± 2.114	−8.509 ± 1.471	0.024 *
***RV Peak Systolic Circumferential Strain Rate (s^−1^)***			
Basal	−0.501 ± 0.050	−0.131 ± 0.037	<0.001 *
Midventricular	−0.484 ± 0.028	−0.349 ± 0.071	0.116
Apical	−0.753 ± 0.162	−0.328 ± 0.084	0.049 *
Lateral	−0.436 ± 0.077	−0.271 ± 0.060	0.128
Septal	−0.723 ± 0.093	−0.269 ± 0.061	0.004 *
Global	−0.579 ± 0.074	−0.270 ± 0.058	0.011 *
***RV Peak Systolic Longitudinal Strain Rate (s^−1^)***			
Basal	−0.647 ± 0.052	−0.586 ± 0.168	0.739
Midventricular	−0.691 ± 0.121	−0.416 ± 0.094	0.111
Apical	−0.500 ± 0.099	−0.125 ± 0.070	0.015 *
Lateral	−0.762 ± 0.103	−0.494 ± 0.103	0.102
Septal	−0.464 ± 0.055	−0.258 ± 0.045	0.020 *
Global	−0.613 ± 0.072	−0.376 ± 0.072	0.049 *
***RV Peak Diastolic Circumferential Strain Rate (s^−1^)***			
Basal	0.379 ± 0.061	0.130 ± 0.049	0.013 *
Midventricular	0.402 ± 0.075	0.346 ± 0.068	0.599
Apical	0.576 ± 0.164	0.236 ± 0.069	0.092
Lateral	0.336 ± 0.110	0.305 ± 0.073	0.819
Septal	0.569 ± 0.091	0.170 ± 0.042	0.004 *
Global	0.452 ± 0.088	0.238 ± 0.055	0.072
***RV Peak Diastolic Longitudinal Strain Rate (s^−1^)***			
Basal	0.454 ± 0.084	0.412 ± 0.115	0.778
Midventricular	0.586 ± 0.180	0.352 ± 0.122	0.312
Apical	0.420 ± 0.103	0.034 ± 0.037	0.008 *
Lateral	0.624 ± 0.151	0.381 ± 0.092	0.206
Septal	0.349 ± 0.081	0.151 ± 0.037	0.057
Global	0.487 ± 0.109	0.266 ± 0.054	0.107

Values are presented as mean ± standard error of mean (SEM).

(*) indicates *p* value < 0.05 for amyloidosis patients compared to controls.

## Data Availability

The data presented in this study are available on request from the corresponding author. The data are not publicly available due to the sensitivity of patient information.
