# Supplementary material for: Biventricular Strain Imaging with Cardiac MRI in Genotyped and Histology Validated Amyloid Cardiomyopathy
Source: Cardiogenetics. Author manuscript; Available in PMC 2021 Sep 1. (PMC8318353; doi:10.3390/cardiogenetics11030011)
Supplement: Supplementary Material [file NIHMS1722379-supplement-Supplementary_Material.pdf]

Table **S1.** Demographics and baseline clinical characteristics of the patients included for histological and imaging analysis.

Patient	Type of presentation	Age/Gender	Race	Hypertension	Diabetes	CHF	LVEF %	Diastolic dysfunction	QRS Duration	LBBB	Amyloid Type
	SOB on Exertion	83 M	Caucasian	No	No	No	63	0	94	No	ATTR
2	CHF exacerbation	78 F	African American	Yes	No	Yes	55	3	90	No	ATTR
3	SOB on exertion	56 M	Caucasian	No	No	Yes	58	2	No EKG	No	AL
4	CHF Exacerbation	78 F	Caucasian	Yes	No	Yes	58	1	148	No	AL
5	SOB on exertion	61 M	Caucasian	No	No	Yes	35	0	99	No	ATTR
6	SOB on exertion	71 M	Caucasian	Yes	No	Yes	58	3	128	Yes	ATTR
7	Acute MI	64 M	Caucasian	No	No	No	43	3	106	No	AL
8	SOB, NSTEMI, CHF	60 F	Caucasian	No	No	No	53	0	90	No	AL
9	SOB, CP	57 M	African American	No	No	Yes	33	0	96	No	AL
10	SOB, edema	83 M	Caucasian	Yes	No	Yes	38	0	108	No	ATTR
11	Weakness	61 M	Caucasian	Yes	Yes	Yes	58	0	88	No	AL
12	DOE	79 M	Caucasian	No	No	Yes	58	3	98	No	ATTR
13	Dyspnea	69 F	African American	Yes	No	Yes	75	3	98	No	AL
14	DOE, edema	62 F	Caucasian	No	No	No	68	0	90	No	AL
15	DOE	85 M	Caucasian	No	Yes	No	58	3	126	No	ATTR
16	Dyspnea	78 M	Caucasian	No	No	Yes	43	0	223	Paced	ATTR
17	LUE weak	84 M	African American	Yes	No	Yes	58	3	161	Yes	Small sample
18	CHF	67 M	African American	Yes	No	Yes	33	2	88	No	ATTR
19	Dyspnea	80 M	Caucasian	No	No	Yes	23	0	87	Paced	ATTR

Abbreviations: CHF - Congestive Heart Failure, CP - Chest Pain, DOE - Dyspnea on Exertion, LUE - Left Upper Extremity, LVEF – Left Ventricular Ejection Fraction, MI - Myocardial Infarction, NSTEMI - Non- ST Elevation Myocardial Infarction, SOB – Shortness of Breath.
